# Occult transgastric fish-bone migration causing delayed left hepatic abscess: serial computed tomography findings and a missed diagnostic opportunity

**DOI:** 10.1093/bjrcr/uaag041

**Published:** 2026-11-02

**Authors:** Xiu-Xiang Liu, Zhi-Xuan Li, Meng Wu, Ming Tian, Hua Ren

**Affiliations:** Department of Radiology, Xiyuan Hospital, China Academy of Chinese Medical Sciences, Beijing 100091, China; Department of Ultrasound, Xiyuan Hospital, China Academy of Chinese Medical Sciences, Beijing 100091, China; Department of Radiology, Xiyuan Hospital, China Academy of Chinese Medical Sciences, Beijing 100091, China; Department of General Surgery, Xiyuan Hospital, China Academy of Chinese Medical Sciences, Beijing 100091, China; Department of Radiology, Xiyuan Hospital, China Academy of Chinese Medical Sciences, Beijing 100091, China

**Keywords:** fish bone, hepatic abscess, foreign-body migration, gastric perforation, computed tomography, ultrasonography, diagnostic error

## Abstract

Ingested fish bones rarely perforate the gastrointestinal tract and migrate into the liver, and the diagnosis may be delayed when ingestion is not recalled and the perforation is locally contained. We report a 45-year-old man who presented with 9 days of intermittent fever, upper abdominal distension, and frequent belching. Ultrasonography demonstrated a large complex collection in the left hepatic lobe with an internal echogenic focus. Non-contrast computed tomography showed a multiloculated left-lobe abscess containing a 5-cm linear hyperattenuating foreign body, focal discontinuity of the gastric antral wall, and hepatogastric inflammatory adhesion. Retrospective review of a chest computed tomography examination performed approximately 4 months earlier showed the same linear foreign body extending from the gastric antrum toward the left hepatic lobe while the adjacent hepatic parenchyma was still normal; the finding had not been reported initially. Laparoscopic drainage yielded approximately 200 mL of pus and enabled intact removal of a 5-cm fish bone, followed by clinical and imaging improvement. This case provides direct serial-imaging evidence of occult transgastric migration preceding hepatic abscess formation. It highlights the importance of reviewing all imaged anatomy, including scan margins, using multiplanar reformations for linear foreign bodies, comparing prior examinations, and identifying and removing a retained foreign body when it is the source of infection.

## Clinical presentation

A 45-year-old man with hypertension was admitted on March 27, 2026 with intermittent fever, upper abdominal distension, and frequent belching for 9 days. His maximum recorded temperature was 38.5 °C. He denied sudden severe abdominal pain, odynophagia, throat pain, or a foreign-body sensation and did not recall ingesting a fish bone. Examination showed right upper-quadrant tenderness and a positive Murphy sign without guarding or rebound tenderness. Laboratory testing demonstrated marked systemic inflammation: white blood cell count 19.43 × 10^9^/L, neutrophils 81.4%, absolute neutrophil count 15.82 × 10^9^/L, C-reactive protein 289.89 mg/L, interleukin-6 96.50 pg/mL, and procalcitonin 0.527 ng/mL.

## Differential diagnosis

In a patient with fever, markedly raised inflammatory markers, and a complex focal lesion in the left hepatic lobe, the principal imaging consideration is a pyogenic hepatic abscess. An infected or necrotic hepatic neoplasm may have overlapping appearances, and the initial right upper-quadrant findings can also direct attention toward biliary infection. In this case, however, the combination of a linear echogenic or hyperattenuating structure, focal gastric antral wall discontinuity, and inflammatory adhesion between the stomach and left hepatic lobe strongly favored a migrated sharp foreign body as the cause of the abscess rather than an incidental internal density.

## Investigations and imaging findings

Abdominal ultrasonography demonstrated an 8.4 × 6.9 × 7.9 cm heterogeneous hypoechoic lesion in the left hepatic lobe containing complex fluid, mobile internal echoes, and a focal echogenic reflector without detectable intralesional vascularity (Figure 1). Non-contrast abdominal computed tomography demonstrated an ill-defined multiloculated low-attenuation collection measuring approximately 21-37 HU and a sharply marginated, toothpick-like linear hyperattenuating structure approximately 5 cm long and 120-160 HU within the collection. Additional findings included surrounding fat stranding, focal discontinuity of the lesser-curvature side of the gastric antrum, and adhesion between the stomach and the left hepatic lobe. There was no diffuse pneumoperitoneum or large-volume intraperitoneal fluid. Sharp fish bones can perforate the stomach or proximal small bowel with minimal free gas because the defect may become locally sealed by adjacent inflammation, omentum, and adhesion.^1–4^

**Figure 1:**
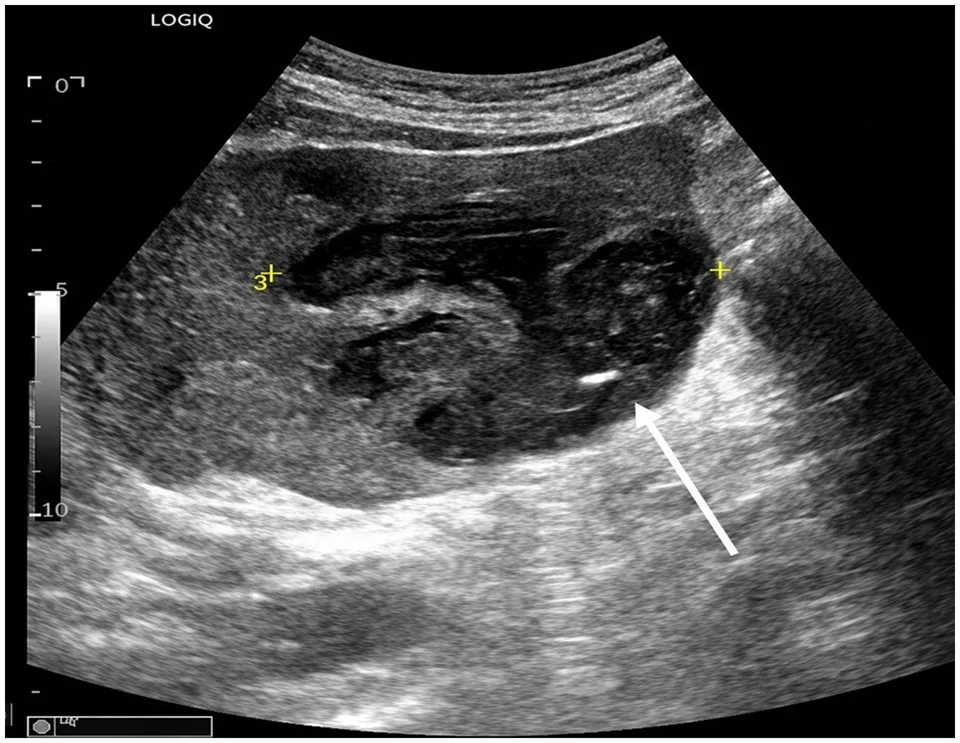
Ultrasonography of the left hepatic lobe on March 27, 2026. Grayscale ultrasonography demonstrates a large heterogeneous hypoechoic collection measuring approximately 8.4 × 6.9 × 7.9 cm, consistent with a hepatic abscess. Complex fluid and internal echogenic material are present. A focal echogenic reflector within the collection (arrow) corresponds to the migrated fish bone in cross-section.

The decisive finding emerged from comparison with prior imaging. A chest computed tomography examination obtained on November 14, 2025 for cough and fever had incidentally included the hepatogastric interface. Retrospective review demonstrated the same linear hyperattenuating foreign body extending from the gastric antrum toward the left hepatic lobe, with no definite hepatic parenchymal abnormality at that time; the finding had not been described in the original report (Figure 2A and B). On the later abdominal examination, the same object was embedded within a newly developed left-lobe abscess (Figure 2C and D). This temporal sequence, together with the focal gastric wall abnormality and hepatogastric adhesion, strongly supported occult transgastric migration with delayed abscess formation. During postoperative re-interview, the patient recalled eating fish before the earlier examination but could not establish the exact ingestion date.

**Figure 2:**
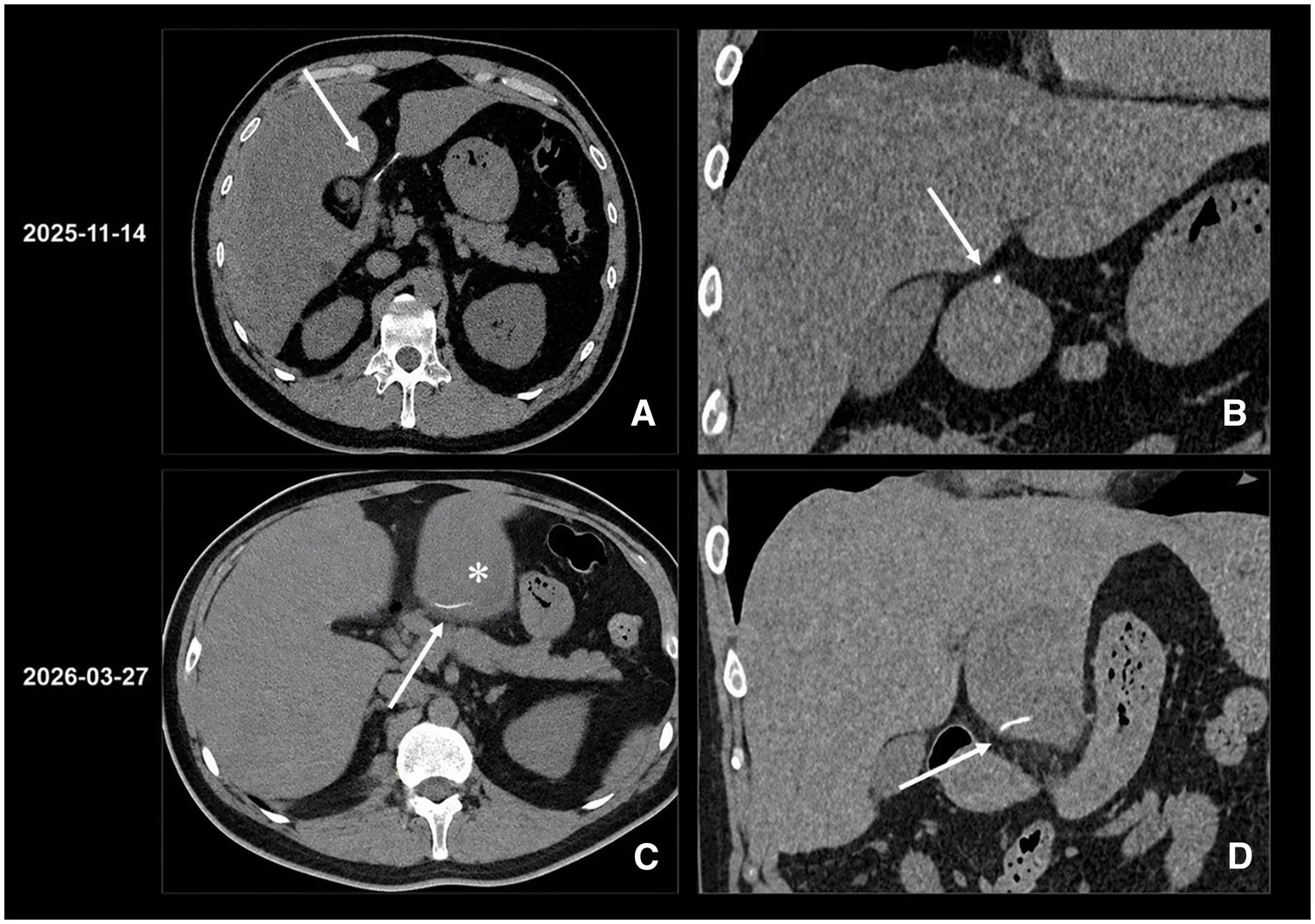
Serial non-contrast computed tomography documenting progression from occult transgastric fish-bone migration to left hepatic abscess. (A) Axial image from November 14, 2025 shows a slender linear hyperattenuating foreign body between the gastric antrum and left hepatic lobe (arrow). (B) Coronal reformatted image shows the foreign body extending from the lesser-curvature side of the gastric antrum toward the left hepatic lobe, without a definite hepatic parenchymal lesion at that time (arrow). (C) Axial image from March 27, 2026 shows the same foreign body within the left hepatic lobe (arrow) and a newly developed low-attenuation abscess (asterisk). (D) Coronal reformatted image confirms the linear foreign body extending from the gastric antral region into the left hepatic lobe in association with the abscess (arrow).

## Treatment

Empirical intravenous cefoperazone-sulbactam and ornidazole were initiated. Emergency laparoscopic exploration demonstrated dense adhesions around the inferior hepatic margin and an enlarged, edematous left hepatic lobe. Needle puncture yielded yellow purulent fluid. Approximately 200 mL of pus was drained from an irregular cavity measuring about 10 × 10 × 8 cm, and a slightly curved 5-cm fish bone was removed intact from the deep portion of the cavity (Figure 3). Two drainage catheters were placed. The gastric wall and suspected perforation site were not directly exposed or repaired during the operation.

**Figure 3:**
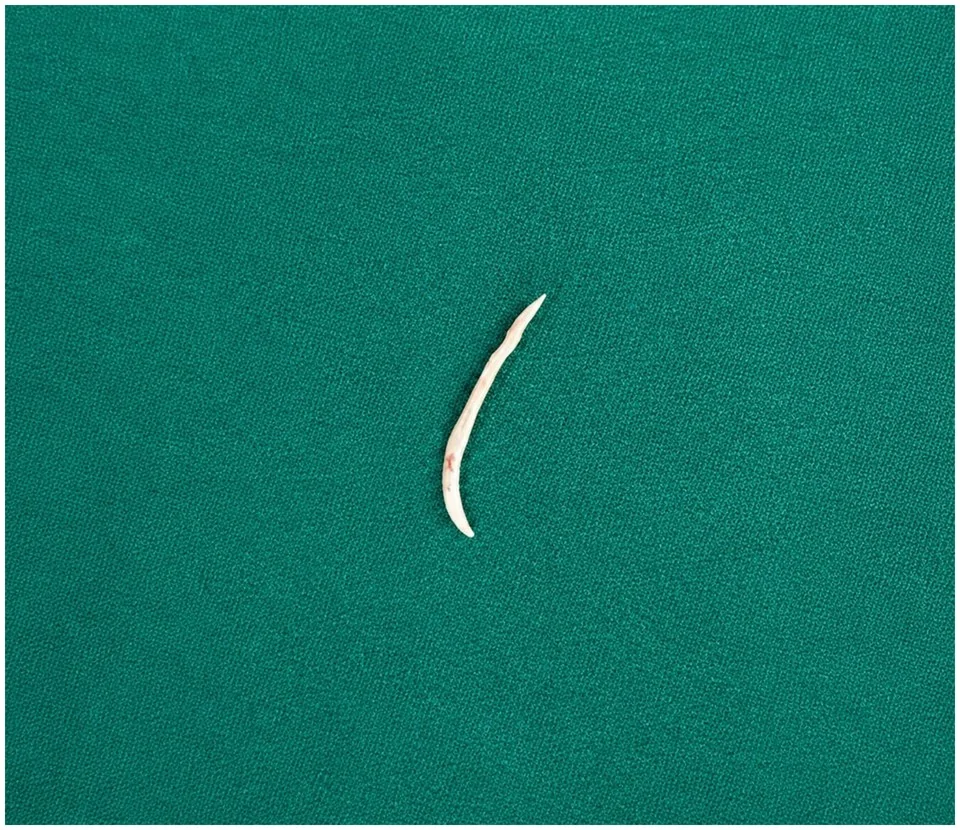
Gross specimen of the retrieved fish bone. Intraoperative photograph shows the slender, slightly curved fish bone removed intact from the deep portion of the left hepatic abscess cavity. The specimen measured approximately 5 cm, corresponding to the linear hyperattenuating foreign body identified on computed tomography.

## Outcome, follow-up, and discussion

After surgery, the patient remained afebrile and improved clinically. By April 2, the white blood cell count had fallen to 15.03 × 10^9^/L, C-reactive protein to 45.27 mg/L, and procalcitonin to 0.183 ng/mL. The drainage catheters were removed on April 4. On May 12, 2026, follow-up computed tomography showed marked reduction of the abscess, and the patient reported no fever, significant abdominal pain, ongoing drainage, or other postoperative complication (Table 1).

**Table 1:** Diagnostic timeline.

Date/period	Clinical event	Diagnostic significance
**November 14, 2025**	Chest computed tomography for cough and fever incidentally included the upper abdomen; a linear foreign body at the hepatogastric interface was not reported, and no hepatic abscess was present.	The causal foreign body was already visible before secondary hepatic abnormality developed.
**9 days before admission**	Intermittent fever, upper abdominal distension, and frequent belching developed.	Symptoms were non-specific and did not reveal the ingestion mechanism.
**March 27, 2026**	Ultrasonography and non-contrast computed tomography demonstrated a left-lobe abscess containing a linear foreign body with focal gastric wall discontinuity and hepatogastric adhesion; antibiotics were started and laparoscopic drainage and extraction were performed.	Imaging linked the abscess, foreign body, gastric wall abnormality, and migration route.
**April 2-4, 2026**	Inflammatory markers declined and drainage catheters were removed.	Clinical response after source control supported the causal role of the retained foreign body.
**May 12, 2026**	The patient remained afebrile and follow-up computed tomography showed marked reduction of the abscess.	Clinical and imaging improvement completed the diagnostic-therapeutic sequence.

Hepatic abscess caused by an ingested fish bone is uncommon. Transgastric migration into the left hepatic lobe is anatomically plausible because of the close relationship between the gastric antrum/lesser curvature and the left hepatic lobe, but patients frequently do not recall ingestion and may not develop a dramatic acute abdominal presentation.^1^^,^^4–8^ Computed tomography is particularly useful because it can demonstrate the foreign body, define its orientation and migration route, identify associated gastrointestinal wall injury, and show secondary inflammatory complications.^1–3^

The distinctive feature of this case is the serial imaging documentation. The causal foreign body was already visible at the hepatogastric interface approximately 4 months before the abscess developed, when the adjacent liver remained radiologically normal. Its location at the caudal margin of a chest examination, outside the primary thoracic search task and without a secondary hepatic abnormality to attract attention, plausibly reduced its conspicuity. Experimental work has shown that even expert observers can miss conspicuous unexpected findings when attention is engaged by another imaging task.^9^ This interpretation should be understood as a diagnostic-safety explanation rather than a reconstruction of the original reader’s cognitive process.

At the later presentation, the key diagnostic step was to treat the linear hyperattenuating structure, focal gastric wall defect, hepatogastric adhesion, and left-lobe abscess as a single causal pattern. The absence of pneumoperitoneum did not exclude perforation, and review of the earlier examination transformed a cross-sectional observation into a temporal migration pathway. Once a retained sharp foreign body is recognized as the infectious nidus, drainage and antimicrobial therapy alone may be insufficient; definitive source control generally requires removal of the foreign body.^5–8^

This report has limitations. The exact date of ingestion was unknown, contrast-enhanced computed tomography was not available, the gastric perforation site was not directly visualized intraoperatively, and microbiological culture results and the complete duration of antimicrobial therapy were unavailable. The transgastric route was therefore inferred from the serial imaging pattern, focal gastric wall discontinuity, hepatogastric adhesion, and operative retrieval of the fish bone rather than confirmed by direct inspection of the gastric defect.

## Learning points

- In an otherwise unexplained left-lobe hepatic abscess, a linear echogenic or hyperattenuating focus should prompt targeted review of the gastric antrum, proximal duodenum, and hepatogastric interface for a migrated sharp foreign body.
- The absence of pneumoperitoneum or generalized peritonitis does not exclude gastrointestinal perforation by a sharp foreign body because local inflammation and adhesion may seal the defect.
- Thin-section computed tomography with multiplanar review, deliberate inspection of anatomy at scan margins, and comparison with prior examinations can reveal a migration pathway that is not apparent on a single study.
- When a retained foreign body is the infectious nidus, definitive source control requires extraction of the foreign body in addition to abscess drainage and appropriate antimicrobial therapy.

## Data Availability

All data relevant to this case are included in the article. Additional deidentified information may be available from the corresponding author on reasonable request, subject to patient privacy and institutional requirements.
